# Supplementary material for: Metagenomic binning with assembly graph embeddings
Source: Bioinformatics. 2022 Aug 16;38(19):4481–7. doi: 10.1093/bioinformatics/btac557 (PMC9525014; doi:10.1093/bioinformatics/btac557)
Supplement: btac557_supplementary_data [file btac557_supplementary_data.zip › amber outputs.zip!/amber outputs/strong100/genome/boxplot_purity_bp.pdf]

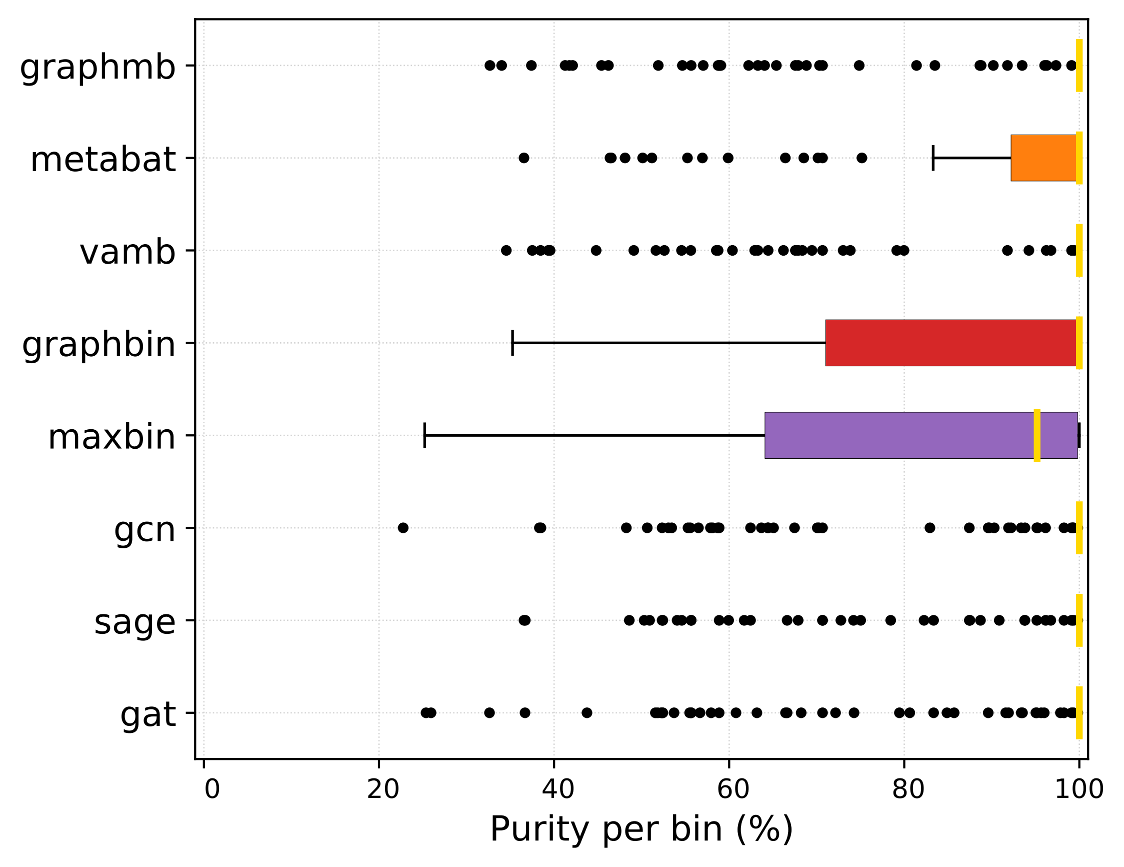
graphmb
metabat
vamb
graphbin
maxbin
gcn
sage
gat
0
20
40
60
80
100
Purity per bin (%)
